# Can popular films instil carcinophobia? Images of cancer in popular Polish cinema

**DOI:** 10.3389/fonc.2022.1062286

**Published:** 2022-12-07

**Authors:** Jan Domaradzki

**Affiliations:** Department of Social Sciences and Humanities, Poznan University of Medical Sciences, Poznań, Poland

**Keywords:** cancer, cancer education, cancer literacy, carcinophobia, cinema, cinemeducation, popular culture, popular films

## Abstract

**Introduction:**

Although cancer is currently considered a serious socio-medical challenge and health education in Poland has been positioned as a public health priority, the impact of popular culture on people’s ideas about cancer has been neglected. This study therefore aims to analyse the way popular Polish films portray cancer and the experience of cancer.

**Material and Methods:**

Seven popular Polish films featuring cancer were analysed both quantitatively and qualitatively. The main categories included in the coding frame were disease, therapy, patient, physicians/oncologists and psychosocial issuses related to cancer.

**Results:**

Polish films fail to provide the audience with basic information about the disease, its diagnoses and treatment and cancer is often represented as a mysterious disease with an unclear cause, an unpredictable and unsuccessful course of treatment, characterised by pain, suffering and inevitable death. Films may therefore instil carcinophobia. Since films accurately reflect problems of daily life faced by cancer patients and their families they have educational potential.

**Conclusion:**

Although Polish films reinforce harmful stereotypes about cancer, its treatment, oncological institutions and specialists, cinema has the ability to raise the public’s and health professionals’ awareness regarding the psycho-social and emotional strains faced by cancer patients and the medical problems related to cancer.

## Introduction

Although advances in medical research have improved the diagnosis, treatment and prognosis of cancer over the years, significantly increasing the cancer patients’ chances of survival, cancer remains the second leading cause of death in OECD countries, accounting for 24% of all deaths in 2019 (1). Poland is among those countries which report the highest rates of morbidy and mortality from cancer. In the last 30 years the number of new cancer cases in Poland has doubled and in 2019 alone the Polish National Cancer Registry registered 171 218 new cases of cancer (85 559 in men and 85 659 in women) and 100 324 deaths (54 371 in men and 45 954 in women) (2). Moreover, even though the overall cancer morbidity rates for both men and women in Poland are lower than the EU averages, the mortality rates are 30% higher for men and 25% higher for women, indicating problems with timely diagnoses and treatment (3). In fact, with 228 deaths per 100 000 Poland’s mortality cancer in among the highest in all OECD countries (1).

One reason for this is that Polish society still has insufficient knowledge about cancer and oncology, including certain risk factors that may increase the incidence of cancer and health behaviours in the population (4, 5). However, peoples’ cancer-related health behaviours are also shaped by lay beliefs, i.e., popular concepts on illness, its meaning and causes, severity of symptoms and appropriate treatment, that arise both from broader theories of illness (e.g., biomedical etc.) as well as social and cultural concepts about the body, health, and illness (6). While lay beliefs can positively influence health-related behaviours, including seeking biomedical treatment, they can also affect people’s pessimistic and fatalistic ideas about cancer and its course (7–9), as well as their opinions about the accuracy and value of cancer screening (10, 11), and discourage the sick from contacting medical professionals.

Simultaneously, although people’s knowledge about cancer has improved lay beliefs about cancer are also greatly influenced by the greater culture at large (12, 13). Moreover, there remain some common myths associated with cancer, such as the belief that cancer is contagious; that it is an untreatable, cruel, slow and lethal disease; that there are no preventive measures for reducing cancer risk, that cancer is a punishment from God, possibly for some sinful behaviour (i.e. infidelity); that cancer treatment is worse than the disease itself; that cancer always hurts; or that cancer ‘should not be touched’ and that the surgery causes cancer to spread (14–19). Unsurprisingly, cultural beliefs about cancer have been acknowledged as important determinants of cancer prevention, controlling behaviours and influencing psychological and behavioural outcomes following cancer diagnosis and treatment (20–23).

Research shows that, while culture plays an important role in shaping people’s lay beliefs regarding cancer, such ideas may lead to needless worrying or may even instil carcinophobia, i.e., a chronic fear related to cancer that manifests itself through feelings of sadness, anxiety, panic, and distress of developing cancer that can, in turn, inhibit individuals from engaging in screening tests, prevention behaviours, and sound treatment decisions (24–26). For example, it was demonstrated that fatalistic beliefs about cancer result in greater fear of cancer and can cultivate cancer stigma (8, 9, 26). Similarly, Synowiec-Piłat showed that the despondent image of Polish oncology reinforces people’s belief in the curability of cancer and delayed reporting of the disease to a physician (10). Such fear is further reinforced by insufficient psychological help for people with cancer covered by health insurance (27).

Equally important research shows that the vast majority of people derive their ideas about cancer from other sources, primarily the media and popular culture, including films (12–14, 28, 29). This is crucial because, even though conventional wisdom and popular culture may perpetuate certain myths and common misconceptions about cancer, its causes and treatment (19, 30–40), these images indisputably constitute an inherent dimension of the social understanding of cancer and are as important for cancer education as the actual science (9, 13).

The Polish oncological community therefore published a *Strategy for Combating Cancer in Poland 2015–2024*, which includes the idea of developing and promoting public education regarding the risk factors for cancer (41). Importantly, apart from launching national social media campaigns, it is also suggested that filmmakers and TV producers should be involved in this, since popular culture is a symbolic resource and a unique “guide” that helps the public to make sense of illness, present cancer research responsibly and can play a crucial role in the public’s construction of ‘cancer imagery’ (42).

Thus, this study aims to analyse the way cancer is depicted in Polish feature films made for theatrical release. While the study was primarily focused on the dominant depictions of cancer in films, it was also interested in the following questions:

1. Do films provide the audience with a knowledge on the symptoms, etiology, and treatment of cancer?
2. How do films portray cancer patients?
3. What images of physicians and healthcare professionals emerge from films?
4. What are the extraclinical, i.e. psychological, social and economic, problems of cancer patients portrayed in films?

## Materials and methods

This study concerns Polish films related to cancer released between 2000 and 2021 inclusively. Although some cancer movies were also produced in Poland in the 1970s and 1980s, the highest interest in cancer among Polish moviemakers can be observed at the start of the new millennium. Moreover, in the year 2000 the first World Cancer Day and the first World Summit Against Cancer was held that year in which the *Charter of Paris Against Cancer* was signed (43). Finally, in June 2000, the Polish Cancer Control Summit was also organised during which the Polish Oncology Union was officially established (44). The sample was designed according to content-based criteria. A film search was conducted in January 2022 using the largest Polish electronic online film database: Filmweb (http://www.filmweb.pl).

The following inclusion criteria were used: 1. they must be feature-length Polish films; 2. the release date must be between 2000 and 2021; 3. the film must be made for theatrical release; 4. the film must portray cancer as the main theme and a character with an explicit cancer diagnosis; 5. the film must be readily available on DVD or online for purchase through Polish streaming sites.

Since this research was limited to feature films in which cancer was the main theme, medically themed television series, i.e. *For good and for bad* [*Na dobre i na złe*], *Physicians* [*Lekarze*], or *Diagnosis* [*Diagnoza*], were ommited. Although they could add something to the discussion about the cinematic portrayal of cancer, as some episodes from these shows featured cancer patients, cancer was frequently only a backstory or was mentioned but remained undeveloped as a complete storyline. Thus, unless the film discussed a character’s cancer story as the main theme and/or the main character had cancer it was not included in the analyses. Additionally, documentaries and shorts films, such as: *Magda, love and cancer* [*Magda, miłość i rak*] or *I want to live* [*Chcę mi się żyć*], were also excluded. While these types of movies might add some diversity to the genres they have been disregarded since the research focused on the way in which popular films depicted cancer. Finally, more pragmatic criteria for film selection were related to their availability on VHS, DVD, or online streaming sites.

To retrieve the relevant films the database was searched using predefined key words: “cancer”, “terminal illness”, “tumour”, “palliative care”. Film titles and available plot descriptions were then scrutinised and compared with the key words and the criteria for inclusion/exclusion. Seven films produced by the Polish film industry between 2000 and 2021 were then selected (**Table 1**). Data from the film database included the year of production, genre and director. In order to make sure that the selected films reached a wider audience, their box office figures were then checked, which, at least to some degree, reflects the scale of their reception.

**Table 1:** List of selected films (N=7).

Film tittle	Type of cancer	Year	Genre	Director	Box office
*The Skylights* [*Jasne błękitne okna*]	brain tumour(glioblastoma multiforme)	2007	Drama	Bogusław Linda	$101,272
*All Will Be Well* [*Wszystko będzie dobrze*]	undefined	2007	Drama	Tomasz Wiszniewski	$59,185
*33 Scenes from Life* [*33 sceny z życia*]	undefined	2008	Drama	Małgorzata Szumowska	$870,504
*Life, Above All* [*Nad życie*]	leukaemia	2012	Drama	Anna Plutecka-Mesjasz	$1,881,974
*Chemo* [*Chemia*]	breast cancer	2015	Drama	Bartosz Prokopowicz	$1,484,357
*Life Must Go On* [*Żyć nie umierać*]	lung cancer	2015	Drama/comedy	Maciej Migas	$967,318
*These Daughters of Mine* [*Moje córki krowy*]	brain tumour(glioblastoma)	2016	Drama/comedy	Kinga Dębska	$3,097,650

Source: http://www.filmweb.pl.

All films that met the inclusion criteria were studied quantitatively and qualitatively. The analysis followed a six-step process which began with becoming familiar with the data, and watching all of the selected films. Next, a standardized and structured data extraction tool was developed which helped to identify important features present in cancer films. Then, the candidate categories were compared and checked against the dataset which helped to determine that they were useful and accurately represented the predefined categories. The final list of categories was created, and a detailed analysis of each film was performed (45, 46). However, during film analysis, some additional attributes were identified and included in the original coding frame. Thus, while the identification of categories included in the coding frame can rely either on the deductive approach, when a researcher is using patterns identified in literature and other studies, or the inductive approach, which aims at identifying new analytic categories that are not limited by existing theories or research, the results from this study were selected by employing a mixed methods approach. Finally, each film was viewed a second time to review all new items. The main categories included in the coding frame were:

- characteristics of cancer (type, aetiology, diagnosis, symptoms, pain, death and dying);
- cancer therapy (diagnosis, type of therapy, medications, response to treatment, side effects, type of care, support);
- characteristics of cancer patients (sex, age, social status, marital status, health behaviour, place and type of death, patient’s self-image and emotional reactions, patients’ problems and needs, family reaction);
- images of physicians (sex, age, specialisation, position, type of researcher, place of work, interest in patient’s disease, communication skills, medical staff);
- psycho-social issues related to cancer (disruption, physical impact, psychological and social impact, medical issues, coping strategies).

These five categories were selected because they describe and identify key points in the scientific literature (30–40). They represent the public’s understanding of cancer in films.

In the last stage of the analysis, all the films were viewed carefully a second time and every scene or passage that supported the pre-determined categories mentioned above was noted on the coding sheet. To achieve this both verbal and non-verbal messages were taken into account. Main categories included in the coding frame and themes were organized through an iterative viewing process based on qualitative research methods and (visual) grounded theory methodology (47, 48). After comparing all notes from all the films, repetitive patterns were found and analysed. Analysis of the qualitative content was also designed to identify recurring patterns in the cinematic images of cancer. To ensure validity of findings an independent researcher specialized in qualitative research was hired to review the coding process. While both researchers reached 93% agreement, the additional peer debriefer helped to resolve the remaining 7% to reach full consensus.

## Results

### Cinematic depictions of cancer

Although most of the films analysed introduced cancer by name (5) many gave only a very scant description of the disease or provided no such information whatsoever (**Table 2**). Frequently the explanation of the cancer consists of no more than a couple of sentences expressed in medical jargon and rests on simplifications. For example, none of the films analysed described the etiology of the cancer. Neither do they provide a description of the symptoms of the cancer. Moreover, although the films depict a variety of cancer symptoms, it was pain that was framed as the most common symptom (5).

**Table 2:** Characteristics of Cancer in the Films.

	N
Mentions the name/type of cancer	
Yes	5
No	2
Type of cancer	
Breast cancer	1
Lung tumour	1
Leukaemia	1
Brain tumour	2
Other, undefined	2
Aetiology of disease	
Genetic	0
Lifestyle	0
Genetic and lifestyle	0
Unmentioned	7
Diagnosis*	
Biopsy	2
Blood test	2
Computed tomography	2
Unmentioned	4
Describing/explaining symptoms	
Yes	0
No	7
Symptoms featured in films*	0
Lump or area of thickening that may be felt under the skin	1
Problems with mobility	1
Breathing difficulties	1
Muscle weakness/Muscle or joint pain	1
Skin conditions	5
Pain	0
Bleeding or bruising	1
Spitting blood	0
Fatigue	2
Loss of weight/inability to gain weight	3
Changes in appetite	2
Nausea	0
Hair loss	0
Loss of concentration	0
Loss of consciousness	2
Loss of speech	2
Memory loss	0
Weakness	3
Dizziness	2
Mood swings/Depression	3
Suffering/Pain	
Yes	5
No	2
Prognosis	
Survives	0
Dies	6
At home	1
In hospital	5
Unspecified	1
Type of death	
Natural death	6
Painful	1
Peaceful	5

*Some appeared in more than one film.

The films studied rarely provide the audience with credible scientific information about diagnostic methods, as blood tests, biopsies and computed tomography appeared in only three of the films, while four mention no diagnostic process at all.

While two films neglect to mention the type of cancer portrayed in the film, brain tumour appered twice, and breast cancer, lung cancer and leukaemia appeared in one film apiece. Of all seven patients portrayed in the films six die and one is presumed dead. Of those who died, five passed away in hosptial and one died at home. Although the death of all the patients was caused by the disease itself, only one died in pain and the five other patients died peacefully.

### Cancer patients in Polish films

Cancer patients portrayed in popular Polish films were predominantly middle aged (6), middle class (4) and women (5) (**Table 3**). Five of the characters lived in a big city and two lived in the country or small town (4). Four were married, one widowed and one divorced. While few looked after themselves either by doing sport or having a healthy diety (one apice) or having medical check-ups (2), almost half were heavy smokers or heavy drinkers (three apiece).

**Table 3:** Patients and their families.

	N
Sex	
Male	2
Female	5
Age	
Child	0
Adolescent	0
Adult	0
Middle-age	6
Elderly	1
Social status	
Higher class	2
Middle class	4
Lower class	1
Domicile	
Country/small town	2
Middle town	0
Big city	5
Marital status	
Single	0
Cohabiting	0
Married	5
Widowed	1
Divorced	1
Lifestyle choices*	
Smoker	3
Drinker	3
Unhealthy diet	0
Sport	1
Medical check-ups	2
Patients’ reactions*	
Shock and disbelief	2
Denial	1
Fear and anxiety	2
Panic attacks	1
Anger, guilt and blame	3
Sadness	0
Depression	2
Fatalism	0
Loneliness	4
Loss of control	1
Hope	5
Acceptance	5
Struggle*	
With cancer	6
With bureaucracy	1
With medical staff	2
Family reaction*	
Care/support	5
Disinterest	2
Denial	1
Anger	2

*Some appeared in more than one film.

Although most characters accepted their disease and their inevitable death (5), cineamtic patients experienced a variety of disheartening emotions, including loneliness (4), anger, guilt and blame (3), shock and disbelief, fear and anxiety or depression (two apiece).

While six of the characters struggled with the disease, two pregnant women also struggled with the condescendingly paternalistic attitudes of their physicians, who insisted on abortion and criticised their eventual decisions to go full term. Five of the patients expereinced love, care and support from their loved ones, while two families displayed a lack of interest or anger and one denied the disease.

### Cancer therapy in films

The majority of films in the study failed to mention diagnostic methods (4). Three of the films, however, depicted blood tests, biopsy and computed tomography (**Table 4**). While chemotherapy and surgery were the most common forms of therapy (five apiece) analysed films minimized the importance of the psychosocial dimension of care for cancer patients, as palliative care was mentioned only once. Simultaneously, traditional Vietnamese medicine appeared twice and in one of the films the patient’s family asked a witch folk healer for help.

**Table 4:** Cancer therapy in films.

	N
Diagnosis*	
Blood test	2
Biopsy	2
Mammography	0
Magnetic resonance imaging	0
Radiography	0
Computed tomography	2
Genetic test	0
Fails to mention	4
Therapy*	
Drug therapy	1
Chemotherapy	5
Radiotherapy	0
Surgery	5
(Bone marrow) transplantation	1
Antalgic therapy/pain-relief	2
Psychotherapy/psycho-oncology	0
Complementary therapy/non-medical healing	
Traditional Vietnamese medicine	2
Falk healer	1
Medications	
Yes	4
No	3
Response to treatment	
Yes	1
No	4
Fails to mention	2
Side effects*	
Pain	3
Muscle or joint pain	3
Changes in appetite	2
Nausea	1
Weight loss, including unintentional weight loss or weight gain	4
Changes in appearance	5
Hair loss	5
Skin condition**s**	3
Persistent cough or breathing difficulties	0
Difficulty in swallowing	0
Bleeding or bruising	1
Reproductive issues	0
Fatigue	4
Cognitive and memory changes	2
Loss of speech	2
Type of care	0
Homecare	0
Hospital care	0
Home and hospital care	7
Hospice/palliative care	1
Support*	
Non-professional internal	
Parent	2
Partner	3
Child	3
Non-professional external	
Friend	3
Neighbour	0
Other patient	0
Professional medical	
Oncologist	2
Physician	2
Nurse	2
Professional psycho-social	
Psychologist/psychiatrist	1
Social worker	0
Priest	4

*Some appeared in more than one film.

While four characters failed to respond to treatment, all patients suffered side effects, including changes in appearance (5), such as hair loss (5), skin conditions (5) or weight loss (4), fatigue (4) or pain (3).

While all the cancer patients received both outpatient and inpatient care, the dominant form of support received came from non-professionals, mainly partners (3), children (3), friends (3) or parents (2). Catholic priests provided spiritual care for the patients in four of the films but psychologist appeared only in one film. Of all twenty eight members of medical staff portrayed in the films only six (two oncologists, two general physicians and two nurses) provided any kind of emotional support, while the rest focused on providing biomedical information regarding patients’ medical condition or therapy.

### Cinematic depiction of physicians

In total, 28 health professionals were identified in films, including nineteen physicians, seven nurses and two electroradiology technicians. Significantly, the stereotypical physician portrayed in the films was middle-aged (18) and male (12) (**Table 5**). From the nineteen physicians, six were oncologists, while five were general practicioners. Nine were portrayed as leaders or chief physicians. All physicians worked in public hospitals. Fourteen physicians showed either no interest in their patient or engaged in some form of insensitive or unprofessional behaviour.

**Table 5:** Cinematic images of physicians.

	N
Sex	
Male	12
Female	7
Age	
Young	0
Middle Age	18
Older	1
Specialisation	
Oncologist	6
Haematologist	1
GP	7
No information	5
Position	
Leader/chief physician/scientist	9
Assistant	0
Not mentioned	10
Place of work	
University hospital	0
Public hospital	19
Private hospital	0
Interest in patient’s illness	
Care/support	5
Lack of interest	2
Insensitive behaviour	12
Medical staff*	
Physician	19
Nurse	7
Electroradiology technician	2

*Some appeared in more than one film.

### Psycho-social issues related to cancer

The qualitative analyses identified six major themes with several associated sub-themes: 1. medical issues; 2. physical impact; 3. psychological impact; 4. social impact; 5. struggle; and 6. coping and support (**Table 6**).

**Table 6:** Main theme.

Theme	Sub-theme	N	Films Illustrating the Theme
Medical issues	Lack of correct diagnosis	1	*All Will Be Well*
	Lack of information	3	*All Will Be Well*; The Skylights; *Chemo*
	Lack of appropriate quality healthcare	2	*All Will Be Well*; *Chemo*
	High cost of drugs and care	2	*All Will Be Well*; *Chemo*
	Inequities in availability of treatment and care	5	*All Will Be Well; The Skylights; The Skylights*; *Life Above All*; *Chemo*;
	Lack of psychological support	6	*All Will Be Well; The Skylights*; *33 Scenes from Life*; *Life, Above All*; *Chemo*; *These Daughters of Mine*
	Side effects of therapies	6	*The Skylights*; *33 Scenes from Life*; *The Skylights*; *Life, Above All*; *Chemo*; *These Daughters of Mine*
	The relationship between the patient and the medical staff	6	*All Will Be Well*; *33 Scenes from Life*; *The Skylights*; *Life, Above All*; *Chemo*; *These Daughters of Mine*
Physical impact	Physical changes	5	*All Will Be Well*; *33 Scenes from Life*; *The Skylights*; *Chemo*; *Life Must Go On*
	Pain	5	*The Skylights; All Will Be Well*; *33 Scenes from Life*; *Life, Above All*; *Chemo*
	Sexuality	1	*Chemo*
Psychological Impact	Identity/self-stigmatisation	4	*The Skylights*; *Life, Above All*; *Chemo*; *Life Must Go On*
	Meaning of life and death	5	*33 Scenes from Life*; *The Skylights*; *Life, Above All*; *Chemo*; *Life Must Go On*
	Fear of death	4	*All Will Be Well*; *33 Scenes from Life*; *Chemo*; *Life Must Go On*
Social Impact	Stigmatisation	2	*All Will Be Well*; *Chemo*
	Isolation/social exclusion	3	*All Will Be Well*; *33 Scenes from Life*; The Skylights
	The economic implications of therapy	2	*All Will Be Well; The Skylights*
	Disruption of relationship between the patient and partner	5	*All Will Be Well; The Skylights*; *Life, Above All*; *Chemo*; 33 Scenes from Life
	Disruption of family dynamics	5	*All Will Be Well*; *33 Scenes from Life; Life, Above All*; *Chemo*; *These Daughters of Mine*
	Loss of job	1	*Life, Above All*
	Reduced life opportunities	3	*Chemo*; *Life, Above All*; *These Daughters of Mine*
Struggle/war/fight	With cancer	6	*All Will Be Well*; *33 Scenes from Life*; *The Skylights*; *Life, Above All*; *Chemo*; *These Daughters of Mine*
	With bureaucracy	2	*Life, Above All*; *Chemo*
	With the medical staff	2	*Life, Above All*; *Chemo*
Coping/support	Family	6	*All Will Be Well*; *33 Scenes from Life*; Skylights; *All Will Be Well*; *Chemo*; *These Daughters of Mine*
	Priest/Religion and faith	2	*All Will Be Well*, *Chemo*
	Professional medical	1	*Life, Above All*
	Professional psychosocial	1	*These Daughters of Mine*

*Most of the films covered more than one theme.

Films featuring cancer stressed patients’ problems in obtainng medical information (3), access to appropriate healthcare and the high costs of drugs and care (two apiece) or the lack of access to correct diagnoses (1). The side effects of cancer therapy, lack of psychological support and problems with their relationship with medical staff were highligthed in six films.

Regarding the physical aspects of cancer, most films focused either on the physical changes caused by cancer (i.e. hair loss, skin conditions or weight loss) or pain (5 apiece). One film stressed the impact of cancer on sexuality.

The most important psychological issues related to cancer referred to patients’ struggle to find meaning as they confronted death (5) and the fear of death itself (4). Four of the films showed the way cancer affects one’s identity or leads to self-stigmatisation.

Most of the films focused on the way cancer disrupts patient’s relationship with partners (5) and the way it damages family dynamics (5). Some showed the way the patient experiences social isolation, exclusion (3) and stigmatisation (2), while others focused on the way cancer reduces one’s life opportunities (3), including the loss of job.

While patients’ struggle with cancer was the leading trope in the films under examination (6), some films also explored each character’s struggle with medical bureaucracy and having to deal with uncaring and arrogant healthcare professionals (2).

While all but one of the films framed family as the main source of psychological and emotional support, the films stressed that cancer patients suffer not only from lack of information of the disease or treatment but also the lack of emotional and psychological support from physicians. Thus, the films under examination show the way the psychosocial consequences of the disease damaged self-image, family dynamics and social relations.

## Discussion

Despite medical advances and government’s cancer control strategies the number of cancer patients and deaths in Poland is on the rise, and it is currently the second leading cause of death in Poland, second to cardiovascular disease. Consequently, cancer is now considered a public health priority and the role of health education as an important component of cancer prevention has been recognised (41, 49, 50). However, while health policy often focuses on primary and secondary prevention programmes, the impact of popular culture on people’s beliefs about cancer seems to be neglected. Meanwhile, since health and illness have become a focus of public interest, popular culture, the cinema included, plays an increasingly important role in shaping the social perception of cancer, its prevention and screening and the effectiveness of cancer therapy (9, 41, 42). Especially that cancer is the most common disease portrayed in films, followed by AIDS/HIV and cardiovascular diseases (51). Since cinema both reflects and constructs the social notions about cancer, it is important to know what image of cancer emerges from the films.

This research confirms findings from previous studies suggesting that films reinforce common stereotypes of cancer that prevail in society and may instil carcinophobia (9, 14, 30–40). Most importantly, depictions of cancer in Polish popular films are at odds with the epidemiological data and misrepresent the distribution of cancer in reality. Although the most common types of cancer in Polish women are breast cancer, lung cancer, colorectal cancer, endometrial cancer and ovarian cancer, and in men prostate cancer, lung cancer, colorectal cancer, bladder cancer and stomach cancer (2, 52), most of the films under examination related to such uncommon and aggressive types of cancer as brain tumour or leukaemia.

Rosti et al.’s analysis of 75 films produced in 13 countries between 1939 and 2012 also demonstrated that only five films covered breast cancer, which is the leading cause of death among women cancer sufferers, the cinema tends instead to focus on less common types of cancer, including lymphoma, CNS tumours and leukaemia (33). Similarly, De Fiore et al. (36) showed that, while the deadliest cancers, i.e. prostate cancer, pancreatic cancer, breast cancer, colorectal cancer and lung cancer, are barely represented in films, relatively rare and aggressive types, i.e. leukaemia, lymphomas and brain tumours, predominate in films. Pavisic et al. showed that films dealing with childhood cancers also tend to focus on rare and aggressive variants of cancer which are usually unresponsive to treatment (35).

This is, however, unsurprising because the aim of popular culture is to entertain rather than educate. Clark observes that films prefer “clean cancers”, since they are less physically unattractive and messy, and do not involve sexual organs (39, 40).

None of the films under examination explained the specificity of the cancer portrayed in the film. Consequently, the aetiology of disease remained obscure, as films referred neither to genetic factors nor those related to unhealthy lifestyle. Although some characters smoked or drank heavily, however, the films made no association between such unhealthy behaviours and cancer (*33 Scenes from Life*, *Life Must Go On*). Additionally, most of the symptoms from which the main characters suffered were less attributed to the disease itself than to the side-effects of cancer treatments, chemotherapy in particular. Thus in accord with Drukarczyk et al.’s observation (51), Polish films simplify the experience of cancer, as they often focus on the advanced stages of disease. Thus, the simplified explanation of cancer renders the educational value of the films regarding the clinical aspects of cancer extremely limited.

The mortality rate among the patients portrayed in Polish cinema is also at odds with reality as it is much higher than in reality: in spite of the progress in the treatment of cancer in all the films cancer was explicitly associated with death (32, 33, 35–37). Thus, taking under consideration the scale of reception of such Polish cancer films as: *Life, Above All*, *Chemo*, *Life Must Go On* and *These Daughters of Mine* one can assume that they can reinforce the stereotype of cancer as an incurable and lethal disease (8, 14, 19). This image of cancer as terminal illness is further reinforced by the suggestion that the most common symptom of cancer is pain, usually present in the final stage of disease.

Significanlty, in all but one of the films here (*The Skylights*) death resulting from cancer is framed as a peaceful event: while some of the characters maintain good functional capacity up to the time of death, many films also avoid direct images of death. Thus, the cinema constructs an image of a “right” way to die: the patients tend to spend their final moments in the hospital, but most frequently death occurs in the patient’s sleep without significant physical deterioration, pain or agony. While such depictions of death may seem encouraging, Niemeyer and Kruse are compelling in their suggestion that this represents the cinema’s tendency to silence the act of dying from cancer (38). The cinematic portrayal of death overlooks the finite nature of the physical body and life, and inevitability of death, which is often perceived as a failure, the end and loss.

This study also shows that Polish films focus on the biomedical aspects of cancer care but fail to portray the complexity of caring for cancer patients (32, 33, 35, 37). In particular, although films tend to focus on highly aggressive types of cancer there is a significant absence of psycho-oncology and palliative care (32, 35, 37) which was suggested only in one film. For example in one of the most popular Polish cancer film: *These Daughters of Mine* psychotherapy is reduced to a short conversation with a psychiatrist who diagnoses one of the protagonist’s daughters with depressions and prescribes her antidepressants. Thus, even though Polish cinema aptly depicts real psychological processes related to a patient’s emotional loneliness, social isolation, and psychological distress, most analysed films reinforced popular beliefs that patients experience the disease, go through treatment and hospitalization without any support from healthcare professionals. However, also Pavisic et al. observe that psycho-social support for childhood cancer patients, if present at all, is often reduced to resources already available to families before the cancer diagnosis (35).

The image of cancer patients in films is also far from truthful, as films often prefer younger patients from the upper and upper or middle classes (*The Skylights*, *All Will Be Well*, *Life, Above All*, *Chemo*, *Life Must Go On*) (32, 34, 36, 38). While filmmakers reinforce the stereotype that the patient fails to recover from cancer, cinema often presents cancer patients as weak and sickly, suffering from severe weight loss and unable to lead normal lives. Moreover, films highlight pessimistic emotions experienced by cancer patients, including fear, depression, loss of control, anger and self-stigma. In *33 Scenes from Life*, for example, Lena, who suffers from breast cancer, becomes depressed and covers the walls of her apartment with inscriptions: “I am scared that I am no longer a woman”, “I look like an alien”, “And what if, because of it, I will hate my child?”, “Cancer, you bald fuck!”.

The films also give a poor image of Polish oncology, as cinematic depictions of hospitals and wards reinforce the pessimistic connotations of death and dying. Although recent films incorporate technological advances, some films, i.e. *All Will Be Well*, *33 Scenes from Life* or *Life, Above All*, depict oncology wards filled with suffering and dying patients, and mourning families. Others depict them as dark and gloomy places that resemble a morgue (*The Skylights*).

Moreover, in films dealing with cancer aslo physicians and oncologists are portrayed as unfavourable stereotypes (53–55) either as arrogant or uncaring individuals who are focused on formal procedures and treatment methods, but are insensitive to patients’ emotional and psychological needs and violate many standards of professionalism. In *Life, Above All*, which is based on the life of the Polish volleyball player Agata, who falls pregnant while waiting for a bone marrow donor, three male physicians shout at her and criticise her for being irresponsible, and suggest she should have done more to “avoid such an alternative”.

In *Chemo* all but one physician treat Lena, who also decides to have a baby, even if it will cost her own life, condescendingly and insist on an abortion without so much as asking her opinion. On the other hand, the only physician who supports Lena’s decision during her consultation shows no empathy when she recommends a mastectomy and chemo and is so busy signing and stamping documents that she does not even look at the parents-to-be. When Lena’s partner asks the doctor fearfully what her chances of survival are, Dr. Sowa replies insensitively: “Usually they survive the delivery”, and than simply asks: “Have you decided?”. Finally, in *These Daughters of Mine* a female doctor informs one of the protagonist’s daughters about her mother’s death, while they are both washing their hands in the toilet. All those examples support Franchina et al’s observation (56) that showed that in films dealing with cancer doctors focus on biomedical aspects of cancer and rarely assume the role of caregivers.

## Limitations

Since the entire number of Polish films depicting cancer cannot be adequately determined, the selection of films cannot be representative. Moreover, because this study was limited to popular feature-length films dealing with cancer, only seven films were included in the analysis. It would therefore be desirable to extend the analysis and compare the content of popular films with different video formats that were omitted here. Since this research was interested in more recent films, it would be also desirable to compare earlier films to ascertain whether and in what way cinematic depictions of cancer have changed over time. Since this study focused on Polish films, it would be also illuminating to extend future analyses to, European, American and Asian cinema. Finally, since this study focused on the ways in which films depict cancer, it is unclear whether and to what extent cinematic representations influence viewers’ cancer-related health behaviours. Thus, further studies examining the impact of watching oncomovies on the risk of developing carcinophobia and cancer awareness among patients and general audiences should be performed.

Despite these limitations, however, there are some advantages to this study. Most importantly, to the best of my knowledge, this is the first study on cinematic depictions of cancer in Polish films. Thus, this study fills a gap in the literature and may stimulate further research into the images of cancer in Polish popular culture. Additionally, since it shows that Polish films both reflect and (re)construct the social images of caner, it may provide a framework through which films may be utilised in teaching providers in oncology about the cultural significance of cancer and the bio-psychosocial complexities of the cancer experience.

## Conclusions

This research highlights the fact that since popular Polish films tend to provide its audience with no basic medical information about the disease, including its symptoms, diagnosis, and treatment, cancer is often portrayed as a far darker disease than it really is. It is represented as a mysterious disease with an unclear cause, an unpredictable and often unsuccessful course of treatment, and is characterised by pain, and suffering. Thus, this research suggests that since Polish feature films tend to reinforce harmful stereotypes about cancer, its treatment, oncological institutions and specialists, Polish cinema might be responsible for the perpetuation of carcinophobia to its audiences. Consequently, while there is an need to increase the public’s cancer health literacy (57, 58), popular movies on cancer can be used to increase cancer awareness among the general public (42, 59–61).

In order to achieve this goal, Polish moviemakers should address negative cancer beliefs, through the destigmatization and normalization of the disease and through the promotion of positive illness trajectory. In particular, information on the need for early detection and treatment should be emphasized. The reason for this being that because cinematic cancer stories strongly influence audiences on an emotional level, watching cancer films can educate the public about the importance and need for cancer prevention and screening (42, 61). Films can also motivate audiences to look for reliable sources of information about the disease and to introduce pro-health changes to one’s life.

Simultaneously, since analysed films accurately reflect daily problems faced by cancer patients and their families, the cinema has the ability to raise the public’s and health professionals’ awareness on the psycho-social and emotional aspects of cancer and medical problems related to cancer, i.e. lack of information about disease, delayed diagnosis, lack of appropriate quality healthcare, the high costs of drugs and care or lack of psychological support from physicians (59–61). Thus, while films may help to educate the public as to the ways of seeing and accepting death from cancer, they may also help overcome the biomedical model of cancer and promote a more holistic approach to the patient.

## Data availability statement

The raw data supporting the conclusions of this article will be made available by the authors, without undue reservation.

## Author contributions

The author confirms sole responsibility for the following: study conception and design, data collection, analysis and interpretation of results, and manuscript preparation.
